# Longet on Phrenology

**Published:** 1844-10

**Authors:** 


*Longet on Phrenology.*—M. Longet admits, as a necessary deduction from the most numerous series of observations and experiments—a deduction borne out by all the facts in our possession whether direct or analogical, that in the cerebral lobes especially exist all the material conditions of the intellect, of the sentiments, and of the instincts; that the number and perfection of the intellectual faculties, in the several species of animals as well as in the individuals of the same species, would appear to be in direct proportion with the extent of the cerebral surface, which is materially influenced by the number and depth of the convolutions; and still further he admits that the doctrine of special and distinct organs, existing in the cerebral hemispheres, appropriated to each faculty of the mind, and to the different moral and instinctive qualities, is neither impossible nor improbable. He at the same time denies that its truth has as yet been demonstrated, and maintains that all attempts that have been heretofore made to localize the different organs of the intellectual, moral, and instinctive faculties have entirely failed.

The modern phrenological doctrines he rejects, and attempts to show that they are entirely unsupported, and even contradicted in many points, by the facts furnished from the pathological conditions of the brain—from direct experiments, as well as by the deductions drawn from comparative anatomy.—*Ibid.*

